# The Impact of Temporal Variation in Indocyanine Green Administration on Tumor Identification During Fluorescence Guided Breast Surgery

**DOI:** 10.1245/s10434-021-10503-2

**Published:** 2021-08-04

**Authors:** Martha S. Kedrzycki, Maria Leiloglou, Vadzim Chalau, Nicolas Chiarini, Paul T. R. Thiruchelvam, Dimitri J. Hadjiminas, Katy R. Hogben, Faiza Rashid, Rathi Ramakrishnan, Ara W. Darzi, Daniel S. Elson, Daniel R. Leff

**Affiliations:** 1 Hamlyn Centre, Institute of Global Health Innovation, Imperial College London, London, UK; 2 Department of Surgery and Cancer, Imperial College London, London, UK; 3 Department of Breast Surgery, Charing Cross Hospital, Imperial College Healthcare NHS Trust, London, UK; 4 Department of Histopathology, Charing Cross Hospital, Imperial College Healthcare NHS Trust, London, UK

## Abstract

**Background:**

On average, 21% of women in the USA treated with Breast Conserving Surgery (BCS) undergo a second operation because of close positive margins. Tumor identification with fluorescence imaging could improve positive margin rates through demarcating location, size, and invasiveness of tumors. We investigated the technique’s diagnostic accuracy in detecting tumors during BCS using intravenous indocyanine green (ICG) and a custom-built fluorescence camera system.

**Methods:**

In this single-center prospective clinical study, 40 recruited BCS patients were sub-categorized into two cohorts. In the first ‘enhanced permeability and retention’ (EPR) cohort, 0.25 mg/kg ICG was injected ~ 25 min prior to tumor excision, and in the second ‘angiography’ cohort, ~ 5 min prior to tumor excision. Subsequently, an in-house imaging system was used to image the tumor in situ prior to resection, ex vivo following resection, the resection bed, and during grossing in the histopathology laboratory to compare the technique’s diagnostic accuracy between the cohorts.

**Results:**

The two cohorts were matched in patient and tumor characteristics. The majority of patients had invasive ductal carcinoma with concomitant ductal carcinoma in situ. Tumor-to-background ratio (TBR) in the angiography cohort was superior to the EPR cohort (TBR = 3.18 ± 1.74 vs 2.10 ± 0.92 respectively, *p* = 0.023). Tumor detection reached sensitivity and specificity scores of 0.82 and 0.93 for the angiography cohort and 0.66 and 0.90 for the EPR cohort, respectively (*p* = 0.1051 and *p* = 0.9099).

**Discussion:**

ICG administration timing during the angiography phase compared with the EPR phase improved TBR and diagnostic accuracy. Future work will focus on image pattern analysis and adaptation of the camera system to targeting fluorophores specific to breast cancer.

Breast conserving surgery (BCS) is currently the cornerstone treatment for early stage breast cancer as, when combined with radiotherapy, it offers equivalent cancer control to mastectomy but with improved quality of life outcomes.^1^ However, BCS is associated with a greater risk of positive resection margins and reoperative intervention.^2^

The scale of inadequate margins can be evidenced through the high national average BCS reoperation rates, reported to be as high as 21.6% in the US^3^ and 27% in the UK.^4^ Positive margins are a major challenge during BCS, as reoperation has negative sequelae to both the patient and healthcare system, resulting in poorer cosmetic outcome, increased psychological burden, delays to neoadjuvant treatment, and increasing treatment costs by approximately $2360 per patient in the US^5^ and £2136 per patient the UK.^6^

In an attempt to decrease re-excision rates, a spectrum of technologies for intraoperative guidance has emerged.^7^ However, limitations include inability to immediately visualize disease at the resection margin, counterintuitive feedback routines, an over-reliance on surgeon interpretation, and/or failure to integrate into the surgical workflow. Optical imaging appears advantageous when compared with existing approaches, providing real-time visual feedback; however, the tumor detection diagnostic accuracy has yet to be proven.^8^,^9^

Fluorescence guided surgery (FGS) is an optical approach that capitalizes on inherent or externally administered fluorescent molecules to identify targeted tissues.^10^ In FGS, the scene is illuminated to excite fluorophores of interest, enabling them to emit light which can then be captured using tailored camera equipment.^10^ The difference between the signal found in the targeted tissue and the surrounding breast tissue can be used to macroscopically demarcate the targeted tissue.^10^

FGS tumor sensitivity depends on the optical system used for image acquisition and the fluorescent probe used. However, current systems require further performance improvements, including compatibility with variable contrast agents, compensation for ambient room light and tissue optical properties, as well as supporting a high spatial resolution, wide working distance, and wide field of view.

The Food and Drug (FDA) approved systems for FGS thus far have mainly used indocyanine green (ICG) as the contrast agent.^11^,^12^ ICG is a well-studied contrast agent which has been approved for clinical use since 1956.^12^ The dye is widely accepted due to its low toxicity profile, excitation and emission spectra range within the near-infrared part of the electromagnetic spectrum, and favorable optical tissue penetration depth.^13^ The strength of its excitation/fluorescence spectral characteristics depend on its concentration^14^ and molecular environment.^15^ In the case of breast tissue, with a systemic injection of 12.5 mg ICG, the emission peak has been reported to be 814 nm.^16^

Clinically approved uses include angiography and lymphography, as ICG is retained within the vessels into which it has been injected (blood or lymph vessels) due to its considerable size upon binding with plasma proteins. Moreover, systemic administration of ICG has recently been investigated for macroscopic tumor fluorescence evaluation in BCS, yet has thus far lacked in sensitivity and specificity.^17^–^19^ This is theorized to be due to extravasation and retention in tumor tissue via penetration of the tumor’s disrupted vasculature and then remaining within the intercellular space^20^ [enhanced permeation and retention (EPR) effect].^21^ Our previous 10-patient BCS feasibility study (REC 18/LO/2018), has shown that our in-house dual camera system detects ICG fluorescence in vivo at a sub-millimeter scale, while fluorescence image texture pattern analysis could improve the tumor detection accuracy.^16^ The primary aims of this follow-on clinical study were to (a) investigate the diagnostic accuracy of ICG fluorescence images for tumor detection during BCS via our developed imaging system, and (b) determine whether the timing of ICG administration affects diagnostic accuracy.

## Methods

Forty patients undergoing BCS were recruited to this single center prospective clinical study approved by a UK Research Ethics Committee (REC 19/LO/0927). The first 10 (07/2020–10/2020) and last 10 (01/2021–02/2021) recruits were allocated to the angiography cohort, and the remaining 20 (10/2020–12/2020) to the EPR cohort. Patients were administered 0.25 mg/kg ICG intravenously (IV), with the EPR cohort receiving the injection before knife to skin, and the angiography cohort receiving the injection once skin flaps were raised. The ICG injection to tumor resection timing was ~ 25 min in the EPR cohort and ~ 5 min in the angiography cohort.

Data was collected on patient demographics (age, height, weight, BMI, ethnicity), tumor and clinicopathological characteristics (size, location, type, grade, hormonal status), operational/ procedural data (time of surgery, time of injection, time of imaging), and procedural outcomes (positive margin rate, reoperation rate, adverse events).

**Fig. 1.**
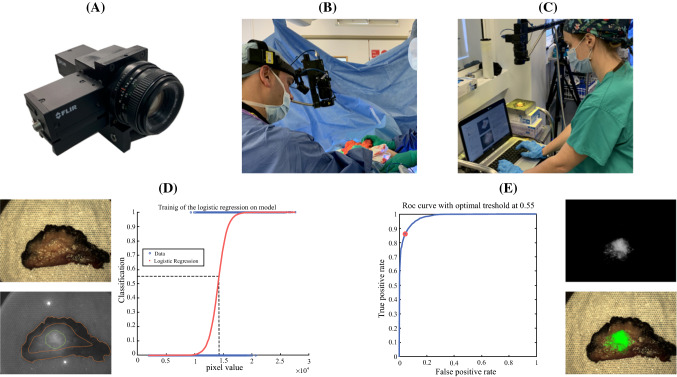
**a** Photographic illustration of the in-house dual camera head fluorescence system (Elson Lab, Imperial College, London).^13^**b** Image acquisition of the tumor in situ. **c** Image acquisition of the excised tumor*.* **d** *Left*: example raw color image (*top*) and fluorescence image (*bottom*) with contouring of tumor (in *green*) and histologically confirmed healthy tissue (between *dotted orange lines*) ground truth regions. *Right*: use of 70% of the contoured ground truth regions to train the classification model. **e** *Left*: use of the remaining contoured ground truth regions to validate the trained model through ROC analysis. In this example, the area under the curve (model accuracy) is 0.98 and when 0.55 [probability for tumor, corresponding to 1.43 × 10^4^ pixel value (*dashed line* in **d**)] is used as the classification threshold the sensitivity and specificity are 0.86 and 0.96, respectively. *Right*: example of processed fluorescence image (*top*), where pixel values below 1.43 × 10^4^ are suppressed to zero, and color image (*bottom*) overlaid with green pseudo-color map indicating probability for tumor upon testing of the trained model across the entire raw fluorescence image

Images were acquired of the tumor in situ prior to resection, ex vivo, of the resection cavity post excision, and during histopathological grossing (Fig. 1a–c). All patients received standard care with surgeons blinded to the fluorescence imaging. Therefore, surgical outcomes were reflective of the conventional techniques being used.

The freshly excised specimens underwent radiography, and subsequently were submitted to fluorescence imaging. Afterwards, they were fixed in formalin, inked, and grossed, at which point histopathologists identified the macroscopic tumor and fluorescence images of each section were taken. The samples were subsequently embedded in paraffin and underwent routine staining and processing. Specimen radiography provided macroscopic information in the en-bloc anterior-posterior view. Although histopathology was able to provide margin information (i.e., 1 mm on superior border), it did not specify at what point along that surface the closest distance occurred, and therefore it could not be correlated to the macroscopic images. Thus, only the excised specimens both en-bloc (anterior and posterior views) guided by both specimen radiograph and histopathology and those obtained during grossing (Fig. 4), were marked for ground truth, whereas the surgical cavity and in vivo tumor images were excluded from analysis.

Pixel values from the tumor and healthy regions for every image were used to calculate the tumor-to-background ratio (TBR) using the below formula:

$${\text{TBR}} = \frac{{{\text{mean}}\,{\text{ pixel }}\,{\text{intensity }}\,{\text{in }}\,{\text{the}}\,{\text{ tumor}}\,{\text{ region}}}}{{{\text{mean }}\,{\text{pixel}}\,{\text{ intensity}}\,{\text{ in }}\,{\text{the }}\,{\text{healthy }}\,{\text{region}}}}.$$

The mean TBR of all the images in each cohort was compared between both timings using the Wilcoxon (non-parametric) test. This analysis was done separately for the ex vivo specimen images (TBR_ex-vivo_) and grossed histopathology images (TBR_histology_). Moreover, for each cohort, TBR analysis was further subdivided based on age, BMI, histological subtype, receptor status and tumor depth.

The Wilcoxon test was employed to determine whether the pixel intensity in the tumor was significantly higher (for *p* < 0.05) than the intensity in healthy regions. This comparison was repeated for each image but also for the pixels from all the images within both cohorts.

Ground truth data was used to train/validate the logistic regression model (Fig. 1d, e). Receiver operating characteristic (ROC) analysis was performed to identify the model’s accuracy in detecting tumor, the optimal classification threshold (pixel intensity above which an image pixel is classified as tumor) and to compute corresponding sensitivity and specificity scores.^22^ Firstly, training and validation was implemented with data from each image separately (image-wise approach). Subsequently, training was performed in all images apart from the image used for validation (leave-one-out cross-validation approach). In both approaches, mean sensitivity and specificity in the two cohorts were extracted separately from the validation scores of all ex vivo and histology images and were used to compare the two different injection timing protocols.

## Results

Forty women were enrolled in this study. Both cohorts were comparable regarding patient demographics and tumor characteristics (Table 1). Preoperatively, one patient had received hormonal therapy and two had received neoadjuvant chemotherapy. Thirteen (32.5%) cases had positive radial margins as defined by the Association of Breast Surgery (ABS) consensus,^23^ with 12 patients (30%) requiring reoperation. There were no drug related adverse events.

**Table 1.** Summary patient demographics and tumor characteristics

	EPR cohort			Angiography cohort		
	Mean	Range (min–max age)	Standard deviation	Mean	Range (min–max age)	Standard deviation
**Patient demographics**						
Age (years), *p* = 0.67	57.9	34–78	±11.7	56.5	33–81	±14.7
BMI (kg/m^2^), *p* = 0.78	25.63^a^	20.32–36.51	±3.96	26.57^a^	19.02–36.6	±4.89
Ethnicity	% (*N*)			% (*N*)		
White-British	40% (8/20)			10% (2/20)		
White-any other white background	10% (2/20)			15% (3/20)		
Black or Black British-African	5% (1/20)			0% (0/20)		
Mixed-any other mixed background	5% (1/20)			0% (0/20)		
Black or Black British-Caribbean	0% (0/20)			5% (1/20)		
Asian or Asian British-Indian	0% (0/20)			5% (1/20)		
Other	40% (8/20)			55% (11/20)		
**Tumor characteristics**						
Size (mm)^b^, *p* = 0.35	13.0	1.7–30	±6.5	15.7	0–34	±9.1
Histological type	% (*N*)			% (*N*)		
IDC	15% (3/20)			15% (3/20)		
IDC + DCIS	70% (14/20)			45% (9/20)		
DCIS	5% (1/20)			15% (3/20)		
ILC ± ISLN	5% (1/20)			15% (3/20)		
IMC + DCIS	5% (1/20)			0% (0/20)		
IMPC + DCIS	0% (0/20)			5% (1/20)		
FAD with atypia	0% (0/20)			5% (1/20)		
Hormone receptor status						
ER+, PR+, HER2−	85% (17/20)			70% (14/20)		
ER+ (DCIS cases)	5 %(1/20)			20% (4/20)		
ER−, HER2 +	0% (0/20)			5% (1/20)		
Triple positive	5% (1/20)			5% (1/20)		
Triple Negative	5% (1/20)			0% (0/20)		
Neoadjuvant treatment						
NACT	5% (1/20)			5% (1/20)		
Hormone therapy^c^	0% (0/20)			5% (1/20)		
Margin status						
Radial positive margins	20% (4/20)			45% (9/20)		
Reoperation rate	20% (4/20)			40% (8/20)		

*IDC* invasive ductal carcinoma, *DCIS* ductal carcinoma in situ, *ILC* invasive lobular carcinoma, *ISLN* in situ lobular neoplasia, *IMC* invasive mucinous carcinoma, *IMPC* invasive micropapillary carcinoma, *FAD* fibroadenoma, *ER* estrogen receptor, *PR* progesterone receptor, *HER2* human epidermal growth factor receptor, *NACT* neoadjuvant chemotherapy

^a^Two patients were excluded from BMI calculation as height was not available

^b^Only invasive cancer was included in the calculation

^c^Hormonal therapy started preoperatively as per local hospital protocol during the peak of the COVID pandemic in patients where surgery needed to be delayed due to theater capacity

Of the anterior and posterior ex vivo images (80 total), 50 were excluded due to an invasive tumor depth > 4 mm (depth penetration limit of ICG fluorescence), therefore 30 TBR values were extracted with a mean TBR of 1.9 (SD ± 0.50). Eight patients were excluded from histopathological grossed image analysis, two due to technical malfunctions, four due to inability to identify the tumor, and two due to no tumor being present (one complete remission post chemotherapy and one false positive of atypia in fibroadenoma). Therefore, 32 TBR values were extracted with a mean TBR of 2.6 (SD ± 1.48).

**Fig. 2.**
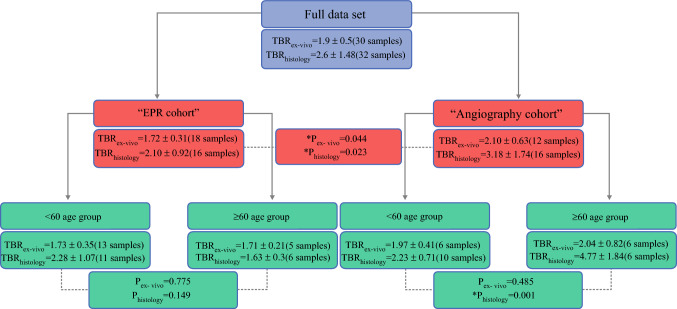
Summary TBR values for the whole dataset, the EPR and angiography cohorts, and sub-groups based on age. Significant differences were found between the two cohorts and between the angiography cohort’s age sub-groups, indicated by *P < 0.05

The graphical synopsis of the TBR comparison results is illustrated in Fig. 2. The TBR for the angiography cohort (ex vivo: 2.10 ± 0.63, histology: 3.18 ± 1.74) was significantly higher than for the EPR cohort (ex vivo: 1.72 ± 0.31, histology: 2.10 ± 0.92) in both ex vivo (*p*_ex-vivo_ = 0.04) and histopathology image analysis (*p*_histology_ = 0.02). There was no significant difference in TBR between the sub-groups of BMI/tumor subtype/depth/receptor status within each cohort, apart from a single age-based sub-group in the grossed histopathology data within the angiography cohort (< 60 years TBR: 2.23 ± 0.71, > 60 years TBR: 4.77 ± 1.84, *p* = 0.001).

**Fig. 3.**
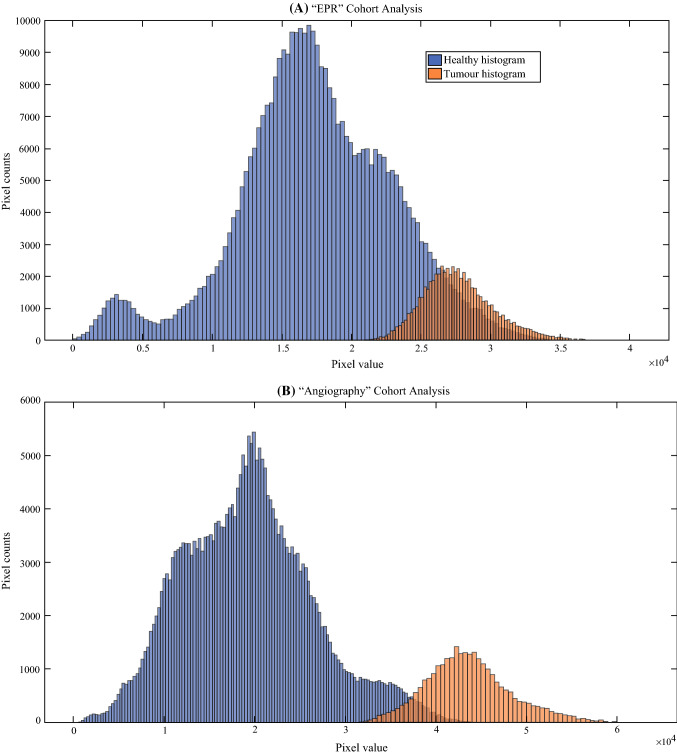
Histograms of tumor regions (*blue*) and healthy regions (*orange*) derived from all images in **a** the EPR cohort and **b** the angiography cohort. A statistically significant difference was observed between the tumor pixel values and the healthy pixel values for **a** (*p* = 0 and *Z* value = 373 and for **b** (*p* = 0 and *Z* value = 274)

**Fig. 4.**
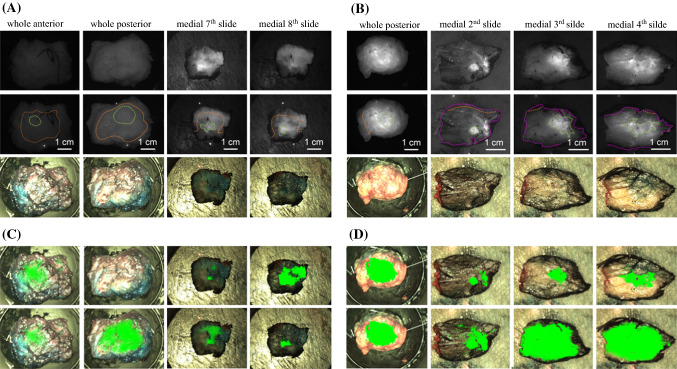
**a**, **b** Examples from ex vivo (after resection) whole specimen and histopathology gross fluorescence images (*first row*) which have been marked (*second row*) for tumor location (*green*), healthy margin (*orange*), orientation-encoding inked edge of specimen (*magenta*) and corresponding color images (*third row*). **c**, **d** Color image overlaid with *green* pseudo-color map, indicating tumor location based on the classification results from the ‘leave-one-out cross-validation’ approach (*first row*) and ‘image-wise’ approach (*second row*). **a, c** are from a single patient in the EPR cohort whereas **b** and **d** are from a single patient in the angiography cohort. In agreement with the validation scores presented in the Results Section, comparison of **c**, **d** (*green* map overlays) with **a**, **b** (ground truth) demonstrates the superior sensitivity of the angiography against the EPR phase

The tumor fluorescence intensity was found to be significantly brighter than healthy tissue in both ‘image-level’ and ‘cohort-level’ analysis (Fig. 3). Sensitivity and specificity scores in the angiography cohort overall outperformed the EPR cohort for both the ex vivo and the histopathology grossed data in both ‘image-wise’ and ‘leave-one-out cross-validation’ approaches, but this was not statistically significant. In the ‘image-wise’ approach, sensitivity and specificity in the angiography cohort were 0.82 and 0.99 (ex vivo) and 0.85 and 0.98 (histology), while the values for the EPR cohort were 0.69 and 0.97 (ex vivo) and 0.72 and 0.93 (histology). In the ‘leave-one-out cross-validation’ approach, sensitivity and specificity in the angiography cohort were 0.80 and 0.88 (ex vivo) and 0.82 and 0.93 (histology), while the values for the EPR cohort were 0.69 and 0.92 (ex vivo) and 0.66 and 0.90 (histology). A series of classification overlays and ground truth contours are depicted in Fig. 4 to demonstrate the technique’s sensitivity and specificity.

## Discussion

The fluorescence signal was significantly superior when IV ICG injection was performed in the angiography phase rather than the EPR phase. This finding discourages acceptance of the EPR phenomenon as the sole contrast mechanism in passive oncological FGS. One possible explanation for the higher TBR values in the angiography cohort could be due to the higher intravascular concentration of ICG that had not yet undergone clearance (reported to be 2–3 min).^24^ Although the difference in TBR was statistically significant between the angiography and EPR cohorts, both achieved clinically acceptable in vivo signals (TBR > 1.5).^25^ Furthermore, administering ICG intravenously at the start of the operation as with the EPR cohort was more easily integrated into clinical workflow.

Since the fluorescence pixel values from the tumor were significantly higher (*p* < 0.001, Fig. 3) than those from the healthy tissue, the logistic regression model was applied to perform an image pixel intensity based classification as recommended by Elliott et al.^26^ Figure 4 overlays and both the “image-wise” and “leave-one-out” validation scores further support the superiority of the angiography versus EPR phase. In the angiography cohort, the sensitivity (0.80–0.85) and specificity (0.88–0.99) demonstrate the potential for clinical translation when compared with prior reports in the literature that describe low sensitivity (0.33) or specificity (0.31).^17^–^19^ However, care must be taken when comparing those scores with the current findings, as they were calculated on a specimen-based rather than a pixel-based classification, as done in the work presented here.

ICG fluorescence could be detected at less than 4 mm from the surface;^27^ therefore, although interrogation depth is sometimes stated to be a limitation of FGS, it is sufficient to establish intraoperatively clear margins during BCS as defined by both the SSO-ASTRO (no ink on tumor for IDC, 2 mm for DCIS)^28^ and the ABS (1 mm for IDC and DCIS) guidelines.^23^ However, given that there was no significant difference in TBR when comparing depth of the tumor, it would be difficult to determine margin thickness. Therefore, ex vivo imaging should be supplemented with a lack of signal in the tumor bed post excision, although leakage from the vasculature or extracellular space may produce false positives.

Electrocautery was used for dissection during BCS for all patients and could result in surgical cavity false positives, potentially affecting the angiography more than the EPR results. This is because in the EPR phase ICG resides in the intercellular space,^20^ whereas in the angiography phase ICG is present within blood due to its binding with plasma proteins,^12^ and therefore would be present if there was any intravascular leakage into the cavity. However, this factor did not affect the validation results presented here, as both in vivo tumor and surgical cavities were excluded from our analysis.

Although localizing techniques have been reported to improve positive margin rates when compared with palpation-guided BCS, they do not completely eliminate the problem.^29^ Many imaging modalities currently exist (radiography, ultrasound, MRI); however, they are either unable to provide detailed intraoperative guidance or require specialist personnel. The benefit of FGS is that it can be utilized intraoperatively via bisection to determine whether tumor-associated fluorescence is seen at the edge of the resection specimen or within the resection cavity, as this would give a high probability that the margin is positive. However, validation of this approach would necessitate comparisons with immediate histological analysis using frozen section. Perhaps the future of BCS will require a combination of macro- and microscopic techniques (e.g., confocal microscopy) to combat positive margins.

Upcoming work will be focused on extracting advanced image texture analysis^16^ in the angiography cohort to exploit the different vasculature characteristics between tumor and normal tissue.^21^ Subsequently, both pixel intensity and texture algorithms will be tested on the remaining images (in vivo, tumor bed, and superior/inferior/medial/lateral ex vivo). Finally, our dual camera FGS system^16^ will be adapted for in vivo imaging using targeting contrast agents more specific to breast cancer.

## Conclusion

The findings of the validation presented here suggest that ICG could be useful for macroscopic tumor evaluation during breast conserving surgery, particularly when administered using short (~5 min), rather than longer (~25 min) intervals for injection prior resection. Although ex vivo results seem encouraging, appropriately powered clinical trials will be required to investigate whether the current regression model can positively impact intraoperative decision-making and patient outcomes.
